# Pythiosis in Africa

**DOI:** 10.3201/eid1103.040697

**Published:** 2005-03

**Authors:** Christine Rivierre, Caroline Laprie, Olivier Guiard-Marigny, Patrick Bergeaud, Madeleine Berthelemy, Jacques Guillot

**Affiliations:** * Clinique Vétérinaire de la Plage, Marseille, France; † Vet-Histo, Diagnostic Histopathologique Vétérinaire, Marseille, France; ‡ Ecole Nationale Vétérinaire d’Alfort, Maisons-Alfort, France Pythiosis in Africa

**Keywords:** Africa, dog, phylogeny, rRNA, pythium, pythiosis, ITS, dispatch

## Abstract

We report the first case of pythiosis from Africa in an 8-month-old dog with a chronic and ulcerative cutaneous lesion. The etiologic agent belonged to the genus *Pythium*. Phylogenetic analysis placed the isolate in a sister group to the other *P. insidiosum* strains. However, the isolate may belong to a new *Pythium* species.

Members of the genus *Pythium* are soil- or water-dwelling organisms that belong to the kingdom Stramenopila (*1*,*2*). More than 200 species of this genus have been described. They usually live as saprophytes, but several species have been reported to cause disease in plants and fish, whereas *Pythium insidiosum* is the only species that has been recognized as a mammalian opportunistic pathogen. *P. insidiosum*–related infection was considered solely an animal disease until 1987, when De Cock et al. reported 2 cases in humans (*3*). In mammals, pythiosis is characterized by the development of cutaneous and subcutaneous abscesses and intestinal necrotic lesions. The bones and lungs may be affected less frequently (*1*). In the absence of specific treatment, pythiosis progresses rapidly, leading to the death of the affected animal or person. To date, the disease has been reported in horses, cattle, dogs, cats, polar bears, and humans (*1*).

The geographic distribution of the disease is very large. Clinical cases have been observed in tropical and subtropical areas of South America (Argentina, Brazil, and Colombia), Central America and the Caribbean islands (Costa Rica, Guatemala, Haiti, Panama, and Nicaragua), North America (the United States, especially in Florida, Louisiana, Mississippi, and Texas), and Asia (India, Indonesia, Japan, New Guinea, New Zealand, North Korea, and Thailand). Recently, evidence for molecular intraspecific variability was demonstrated, according to the geographic origin of 29 *P. insidiosum* isolates (*4*). Pythiosis has not been reported in Europe. The presence of *P. insidiosum* in Africa is likely. In a recent review, Mendoza (*1*) pointed out that “the geographical location and tropical climate of Africa seemingly would make it an ideal region for pythiosis.” Nevertheless, human or animal cases from Africa have not been reported previously.

## Case Report

**Figure 1:**
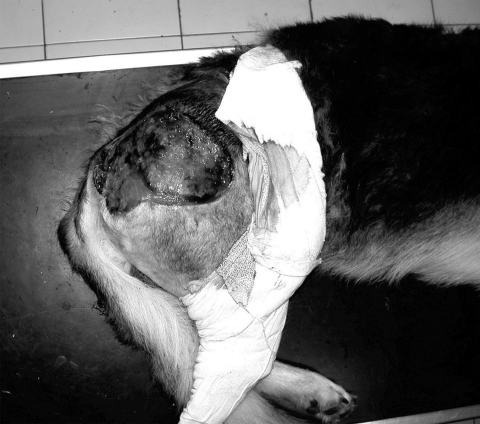
Unique cutaneous lesion on the right side of the hip in an 8 month-old German Shepherd. The lesion had appeared 4 months before this image was taken and had rapidly evolved into a large ulcer with draining tracts.

**Figure 2:**
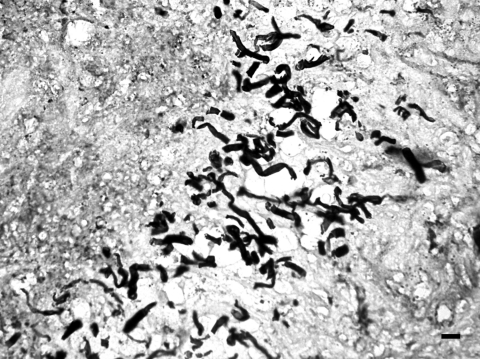
Presence of numerous, broad (3−9 μm in diameter), irregular, septate hyphae in a pyogranulomatous dermatitis (Gomori methenamine silver stain). Bar = 15 μm.

We recently diagnosed a case of subcutaneous pythiosis in an 8-month-old German Shepherd dog originally from Bamako, Mali, a country in northwestern Africa. The dog was born in that region and had lived in Mali until his infection required special treatment. When the cutaneous lesion became chronic and unresponsive to surgical and medical treatments, the owners brought the dog to France for further examination and diagnosis. At the physical examination, the dog had a large (15 cm in diameter), ulcerative and draining, single, cutaneous lesion on the right side of the hip (Figure 1). The animal was otherwise in good health. The cutaneous lesion had appeared in May 2003, and despite several surgical excisions and oral administration of antimicrobial agents, the lesion progressed. In September 2003, several skin biopsy specimens were excised from the lesions. Histopathologic examination showed a pyogranulomatous inflammation with numerous broad (3−9 μm), irregular, septate hyphae (Figure 2). The hyphae were easily observed on Gomori methenamine silver− and periodic-acid Schiff−stained sections, but cultures from biopsy samples failed to grow on Sabouraud dextrose agar.

To obtain a specific identification, genomic DNA was extracted from biopsy specimens collected from the infected tissues (Dneasy Tissue kit, Qiagen, Valencia, CA, USA) and was further subjected to internal transcribed spacer (ITS) and 5.8S rRNA gene sequencing by using primers ITS1 and ITS4 (*5*). Sequencing reaction was performed in a 10-μL volume containing 50 ng of sample DNA, 4 pmol of primers, and 4 μL of BigDye Mix (Applied Biosystems, Foster City, CA, USA). The unique polymerase chain reaction product was analyzed on an ABI Prism genetic analyzer (Applied Biosystems). We obtained a sequence of 785 bp (GenBank accession no. AY683444), which was closely related to ITS and 5.8S rRNA sequences of *P. insidiosum* (GenBank accession no. AY151157-79) (BLASTn, National Center for Biotechnology Information, Bethesda, MD, USA). The dog was treated with oral ketoconazole (10 mg/kg/day) for 5 weeks. However, antimicrobial therapy was not sufficient to shrink the cutaneous lesion to a size that could permit surgery, and the dog was finally killed in December 2003. A necropsy was not performed.

Pythiosis most commonly affects young and large breed (>20 kg) dogs (*6*,*7*). The disease usually occurs in the cutaneous tissue and the gastrointestinal tract. Outdoor and hunting dogs, which are likely to be in contact with swampy water, are at higher risk of contracting pythiosis. The dog in this report was living in a large park and had no access to a swamp. The dog had swum in the Niger River a few weeks before the cutaneous lesion appeared. However, the owners did not recall any trauma, puncture, or wound at the site of the infection.

The climate in Mali is subtropical to arid. Bamako is located in the Sudanese climatic region with an average annual rainfall of ≈55 inches. In that region, the year is divided into 2 major seasons: a cool and dry season from November to February and a rainy season from June to September. The cutaneous lesion of the dog appeared at the beginning of the rainy season.

## Conclusions

**Figure 3:**
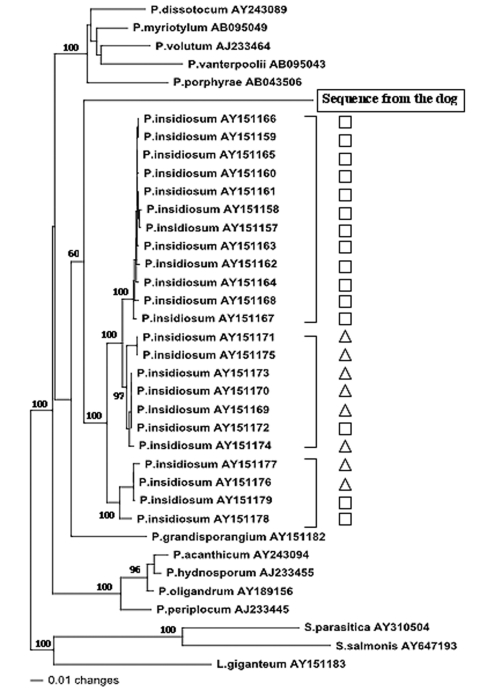
Evolutionary tree of 37 internal transcribed spacer (ITS) and 5.8S rRNA sequences from oomycetes. Each sequence is identified by a Genbank accession number. Shown are 23 sequences from *Pythium insidiosum* from America (□) and Asia/Australia (△). The phylogram presented resulted from bootstrapped data sets (*3*) using parsimony analysis (heuristic search option in PAUP 4.0). This tree was identical to the consensus of 17 most parsimonious trees generated from the branch and bound algorithm in PAUP 4.0. The percentages above the branches are the frequencies with which a given branch appeared in 1,000 bootstrap replications. Bootstrap values below 50% are not displayed. The evolutionary distance between two sequences is obtained by summing the lengths of the connecting branches along the horizontal axis, using the scale on the bottom. *Lagenidium giganteum* (AY151183), *Saprolegnia parasitica* (AY310504), and *S. salmonis* (AY647193) were chosen as outgroups.

To identify more precisely the etiologic agent of the disease, we conducted a complete phylogenetic analysis of the ITS sequence obtained from the dog’s infected tissues and other *Pythium* spp. sequences. Representative ITS and 5.8S rRNA sequences were obtained from GenBank and initially aligned with Clustal X version 1.63b (Institut de Genetique et de Biologie Moleculaire et Cellulaire, Strasbourg, France) and then by visual optimization. To infer phylogenetic relationships among *Pythium* isolates of our dataset, we conducted neighbor-joining and maximum parsimony (*8*) analyses using PAUP 4.0b9 software (*9*). Maximum parsimony analysis was performed by using heuristic searches. Evaluation of statistical confidence in nodes was based on 1,000 bootstrap replicates (*10*). ITS and 5.8S rRNA sequences from other oomycetes (*Lagenidium giganteum*, GenBank accession no. AY151183; *Saprolegnia parasitica*, GenBank accession no. AY310504; and *S. salmonis*, GenBank accession no. AY647193) were chosen as outgroups. The phylogenetic analyses were performed by taking into account 842 characters, including gaps. The total number of 264 characters was constant, 214 were variable but parsimony uninformative, and 364 were parsimony informative. Congruent phylogenetic trees in terms of branching and clustering of taxa were generated with the neighbor-joining (Figure 3) and maximum parsimony methods. In each of these topologies, ITS and 5.8S rRNA sequences from *P. insidiosum* isolates were aligned into 3 clusters previously described by Schurko et al. (*4*). Inside and between these clades, the genetic distances were very low (0.1%–2.8%). The sequence AY683444, corresponding to the canine case, was very different from all other oomycetes sequences (genetic distances varied from 25.0% to 48.0%). Although the dog’s ITS sequence clearly could be grouped with the other *P. insidiosum* clusters, the branching associated this sequence with the *P. insidiosum* strains was supported with only 60% bootstrap values. Sequences from other *Pythium* species formed 2 distinct groups. In the first group (*P. dissotocum*, *P. myriotylum*, *P. volutum*, *P. vanterpoolii*, and *P. porphyrae*), genetic distances varied from 7.1% to 19.9%. In the second group (*P. acanthicum*, *P. hydnosporum*, *P. oligandrum*, and *P. periplocum*), genetic distances varied from 1.8% to 6.2%. This finding suggests that the causative agent of the disease represents a new species within the genus *Pythium*. Because the isolation in pure culture of the etiologic agent was not successful, its ecologic and other characteristics remain to be determined. Specific nutritional requirements might account for the failure of the isolation of this particular strain. This report indicates that more cases of pythiosis in animals or humans from Africa could be expected in the near future.
